# A Pan-European Study of the *C9orf72* Repeat Associated with FTLD: Geographic Prevalence, Genomic Instability, and Intermediate Repeats

**DOI:** 10.1002/humu.22244

**Published:** 2012-10-30

**Authors:** Julie van der Zee, Ilse Gijselinck, Lubina Dillen, Tim Van Langenhove, Jessie Theuns, Sebastiaan Engelborghs, Stéphanie Philtjens, Mathieu Vandenbulcke, Kristel Sleegers, Anne Sieben, Veerle Bäumer, Githa Maes, Ellen Corsmit, Barbara Borroni, Alessandro Padovani, Silvana Archetti, Robert Perneczky, Janine Diehl-Schmid, Alexandre de Mendonça, Gabriel Miltenberger-Miltenyi, Sónia Pereira, José Pimentel, Benedetta Nacmias, Silvia Bagnoli, Sandro Sorbi, Caroline Graff, Huei-Hsin Chiang, Marie Westerlund, Raquel Sanchez-Valle, Albert Llado, Ellen Gelpi, Isabel Santana, Maria Rosário Almeida, Beatriz Santiago, Giovanni Frisoni, Orazio Zanetti, Cristian Bonvicini, Matthis Synofzik, Walter Maetzler, Jennifer Müller vom Hagen, Ludger Schöls, Michael T Heneka, Frank Jessen, Radoslav Matej, Eva Parobkova, Gabor G Kovacs, Thomas Ströbel, Stayko Sarafov, Ivailo Tournev, Albena Jordanova, Adrian Danek, Thomas Arzberger, Gian Maria Fabrizi, Silvia Testi, Eric Salmon, Patrick Santens, Jean-Jacques Martin, Patrick Cras, Rik Vandenberghe, Peter Paul De Deyn, Marc Cruts, Christine Van Broeckhoven, on behalf of the European Early-Onset Dementia (EOD) Consortium

**Affiliations:** 1 Department of Molecular Genetics, VIBAntwerp, Belgium; 2 Institute Born-Bunge, University of AntwerpAntwerp, Belgium; 3 Department of Neurology and Memory Clinic, Hospital Network Antwerp, Middelheim and Hoge BeukenAntwerp, Belgium; 4 Brain and Emotion Laboratory Leuven, Department of Psychiatry, University of Leuven and University Hospitals Leuven GasthuisbergLeuven, Belgium; 5 Department of Neurology, University Hospital Ghent and University of GhentGent, Belgium; 6 Centre for Ageing Brain and Neurodegenerative Disorders, Neurology Unit, University of BresciaBrescia, Italy; 7 Department of Psychiatry and Psychotherapy, Technische Universität MünchenMunich, Germany; 8 Institute of Molecular Medicine, Faculty of Medicine, University of LisbonLisbon, Portugal; 9 Faculty of Medicine, University of LisbonLisbon, Portugal; 10 Department of Neurological and Psychiatric Sciences, University of FlorenceFlorence, Italy; 11 Department of Neurobiology, Care Sciences and Society, KI-Alzheimer Disease Research Center, Karolinska InstitutetStockholm, Sweden; 12 Department of Geriatric Medicine, Genetics unit, Karolinska University HospitalStockholm, Sweden; 13 Alzheimer's disease and Other cognitive disorders unit, Department of Neurology, Hospital ClinicBarcelona, Spain; 14 Biobanc, Hospital Clinic, Institut d'Investigacions Biomèdiques August Pi i Sunyer (IDIBAPS)Barcelona, Spain; 15 Neurology Department, Centro Hospitalar Universitário de CoimbraCoimbra, Portugal; 16 Faculty of Medicine, University of CoimbraCoimbra, Portugal; 17 Center for Neuroscience and Cell Biology, University of CoimbraCoimbra, Portugal; 18 Laboratory of Epidemiology Neuroimaging & Telemedicine & National Centre for Alzheimer's and Mental Diseases, IRCCS Centro San Giovanni di Dio, FBFBrescia, Italy; 19 Department of Neurodegeneration, Hertie Institute for Clinical Brain Research and Centre of NeurologyTuebingen, Germany; 20 German Research Center for Neurodegenerative Diseases (DZNE), University of TuebingenTuebingen, Germany; 21 Clinical Neuroscience Unit, Department of Neurology, University of BonnBonn, Germany; 22 German Center for Neurodegenerative Diseases (DZNE), University of BonnBonn, Germany; 23 Department of Psychiatry, University of BonnBonn, Germany; 24 Department of Pathology and Molecular Medicine, Thomayer HospitalPrague, Czech Republic; 25 Institute of Neurology, Medical University ViennaVienna, Austria; 26 Department of Neurology, Medical University SofiaSofia, Bulgaria; 27 Department of Cognitive Science and Psychology, New Bulgarian UniversitySofia, Bulgaria; 28 Department of Biochemistry, Molecular Medicine Center, Medical UniversitySofia, Bulgaria; 29 Neurologische Klinik, Ludwig Maximilians UniversitätMunich, Germany; 30 Zentrum für Neuropathologie und Prionforschung, Ludwig-Maximilians-Universität MunichMunich, Germany; 31 Section of Neuropathology, Department of Neurological, Neuropsychological, Morphological and Movement Sciences, University of VeronaVerona, Italy; 32 Cyclotron Research Centre and Department of Neurology, University of LiègeLiège, Belgium; 33 Department of Neurology, Antwerp University HospitalEdegem, Belgium; 34 Laboratory for Cognitive Neurology, Department of Neurology, University of Leuven and University Hospitals Leuven GasthuisbergLeuven, Belgium

**Keywords:** *FTLD*, *C9orf72*, repeat expansion, intermediate alleles, European Early-Onset Dementia consortium

## Abstract

We assessed the geographical distribution of *C9orf72* G_4_C_2_ expansions in a pan-European frontotemporal lobar degeneration (FTLD) cohort (*n* = 1,205), ascertained by the European Early-Onset Dementia (EOD) consortium. Next, we performed a meta-analysis of our data and that of other European studies, together 2,668 patients from 15 Western European countries. The frequency of the *C9orf72* expansions in Western Europe was 9.98% in overall FTLD, with 18.52% in familial, and 6.26% in sporadic FTLD patients. Outliers were Finland and Sweden with overall frequencies of respectively 29.33% and 20.73%, but also Spain with 25.49%. In contrast, prevalence in Germany was limited to 4.82%. In addition, we studied the role of intermediate repeats (7–24 repeat units), which are strongly correlated with the risk haplotype, on disease and *C9orf72* expression. In vitro reporter gene expression studies demonstrated significantly decreased transcriptional activity of *C9orf72* with increasing number of normal repeat units, indicating that intermediate repeats might act as predisposing alleles and in favor of the loss-of-function disease mechanism. Further, we observed a significantly increased frequency of short indels in the GC-rich low complexity sequence adjacent to the G_4_C_2_ repeat in *C9orf72* expansion carriers (*P* < 0.001) with the most common indel creating one long contiguous imperfect G_4_C_2_ repeat, which is likely more prone to replication slippage and pathological expansion.

## Introduction

A pathological expansion of a hexanucleotide G_4_C_2_ repeat in the promoter region of the gene *C9orf72* (MIM #614260) was recently identified as the long sought-after underlying gene defect [Dejesus-Hernandez et al., 2011; Gijselinck et al., 2012; Renton et al., 2011] of linkage [Boxer et al., 2011; Gijselinck et al., 2010; Luty et al., 2008; Le Ber et al., 2009; Morita et al., 2006; Pearson et al., 2011; Vance et al., 2006; Valdmanis et al., 2008] and association [Laaksovirta et al., 2010; Shatunov et al., 2010; van Es et al., 2009; Van Deerlin et al., 2010] of frontotemporal lobar degeneration (FTLD) and amyotrophic lateral sclerosis (ALS) to the chromosome 9p21 region [Van Langenhove et al., 2012a]. In the Flanders-Belgian population, we calculated that the pathological G_4_C_2_ expansion is the second most common genetic cause of FTLD [Gijselinck et al., 2012; Van Langenhove et al., 2012b]. Particularly in the subgroup of familial patients presenting with ALS also (FTLD–ALS), *C9orf72* was the first causal gene explaining up to 85.71% of patients [Gijselinck et al., 2012; Van Langenhove et al., 2012b]. Since then, several patient cohorts of different geographical regions have been screened for this repeat expansion mutation establishing *C9orf72* as a major gene for FTLD with frequencies of 7%–11% in total FTLD and 12%–25% in familial FTLD patients [Boeve et al., 2012; DeJesus-Hernandez et al., 2011; Ferrari et al., 2012; Gijselinck et al., 2012; Hsiung et al., 2012; Mahoney et al., 2012; Majounie 2012; Renton et al., 2011; Simon-Sanchez et al., 2012; Snowden et al., 2012].

Different possible disease mechanisms have been proposed including haploinsufficiency and RNA toxicity [Dejesus-Hernandez et al., 2011; Gijselinck et al., 2012] and extensive genotype–phenotype correlation studies are being reported [Al-Sarraj et al., 2011; Arighi et al., 2012; Bigio, 2012; Boeve et al., 2012; Chio et al., 2012; Dejesus-Hernandez et al., 2011; Ferrari et al., 2012; Gijselinck et al., 2012; Hsiung et al., 2012; Majounie et al., 2012; Mahoney et al., 2012; Murray et al., 2011; Renton et al., 2011; Simon-Sanchez et al., 2012; Snowden et al., 2012; Troakes et al., 2011; Whitwell et al., 2012]. However, very little or nothing is known about the mutation spectrum, the genomic mechanism by which the G_4_C_2_ repeat is expanding, and the impact of repeat length on disease susceptibility and gene expression.

In the present study, we aimed at expanding our *C9orf72* observations in the Flanders-Belgian FTLD and FTLD–ALS cohort (*n* = 360) with a larger European cohort of 845 FTLD and FTLD–ALS patients, in which we determined the geographical distribution and prevalence of the pathological G_4_C_2_ expansion. Furthermore, we provide the first evidence for a role of G_4_C_2_ intermediate repeat length on *C9orf72* expression and hypothesize on the genomic mechanisms favoring pathological expansion of the G_4_C_2_ repeat.

## Materials and Methods

### Study Populations

The European FTLD cohort was collected through the European Early-Onset Dementia (EOD) consortium (Supp. Table S1). The European EOD consortium was launched in August 2011 to centralize and harmonize epidemiological, clinical, and biological data together with biomaterial of EOD patients throughout the Europe to stimulate high-profile translational dementia research. Supp. Table S1 describes the number of patients per country and per clinical subgroup contributed by the European EOD consortium members. We received DNA and clinical and demographic information on 917 unrelated FTLD and FTLD–ALS patients as well as histopathology data of 46 patients obtained at autopsy. The 917 patients also included 10 patients from Wallonia, the French speaking part of Belgium, and six more patients from Italy, Spain, and Sweden, which were referred for clinical genetic testing to the Diagnostic Service Facility in our Department of Molecular Genetics (DMG DSF). Patients had been diagnosed according to established clinical diagnostic Neary criteria [Neary et al., 1998] and to the Mackenzie consensus criteria for neuropathology diagnosis [Mackenzie et al., 2010].

The Flanders-Belgian cohort consisted of 337 unrelated patients with FTLD and 23 with FTLD–ALS. These patients were recruited through the Belgian Neurology (BELNEU) consortium, a collaboration with neurologists affiliated to nine different specialized memory clinics and neurology departments in Belgium [Gijselinck et al., 2012; Van Langenhove et al., 2012b] (Supp. Table S2). In addition to the patient cohort, a Flanders-Belgian control cohort was assembled (*n* = 1,083). For more detailed description see Supp. *Materials and Methods*.

### Histopathology of *C9orf72* Expansion Carriers

From 11 *C9orf72* G_4_C_2_ expansion carriers, formalin-fixed brain was available for immunohistochemistry. Five micrometer slices were obtained from frontal cortex, temporal neocortex, hippocampus, area striata, neostriatum, mesencephalon, pons, and cerebellum. Of seven cases, additional samples were provided from thalamus and spinal cord. Slides were stained against Ubiquitin, p62, hyperphosphorylated tau, β-amyloid, TDP43, and FUS. For technical details see Supp. *Materials and Methods*.

### *C9orf72* G_4_C_2_ Genotyping assays

**Figure 1:**
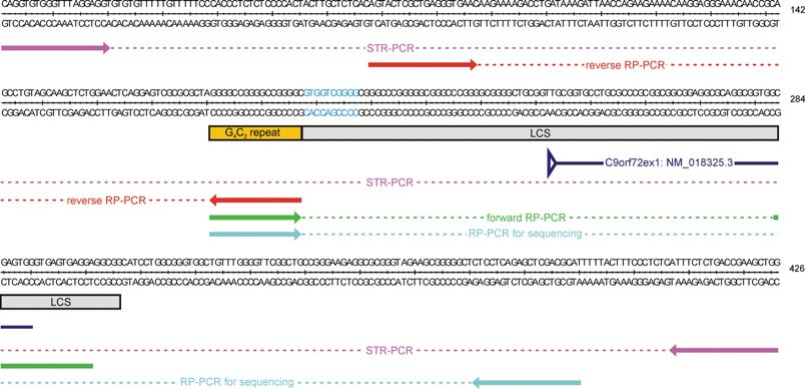
Genotyping assays to characterize the *C9orf72* region and G_4_C_2_ repeat. The *C9orf72* G_4_C_2_ repeat (yellow box) is located upstream of the first exon of isoform NM_018325.3 (dark blue arrow) and adjacent to a GC-rich low-complexity sequence (LCS; light grey box) with their nucleotide sequences shown above. The sequence of the recurrent 10-bp deletion g.26747_26756del**GTGGT**CGGGG (Table 4, Supp. Figure S1), we observed in the LCS, is indicated in blue. Below the sequence, the primers with their corresponding PCR amplicons are shown for each of the PCR genotyping assays: STR-PCR in pink, forward RP-PCR in green, reverse RP-PCR in red and RP-PCR for sequencing in blue.

We developed an alternative repeat-primed PCR assay (reverse RP-PCR; Fig. 1) and a short tandem repeat (STR) fragment length assay with flanking primers optimized for alleles with high GC content (STR-PCR; Fig. 1) allowing reliable identification of G_4_C_2_ expansion carriers and exact sizing of normal lengths. These assays were performed in both cohorts and in relatives of the younger generation of index patients carrying an intermediate repeat allele or a variation in the flanking LCS without expansion.

For technical details on primers and amplification protocols see Supp. *Materials and Methods*.

### Sequencing of the *C9orf72* GC-Rich Low Complexity Sequence

We used the product of an alternative forward RP-PCR (RP-PCR for sequencing; Fig. 1) and sequenced the low complexity sequence (LCS) with the locus-specific reverse primer.

The Flanders-Belgian patient (*n* = 317) and control (*n* = 752) cohorts and 57 expansion carriers and 114 nonexpansion carriers of the European cohort were successfully screened. Cosegregation of LCS variations with the presence of a G_4_C_2_ expansion was analyzed in two available families. For technical details see Supp. *Materials and Methods*.

### *C9orf72* Exon Sequencing and Dosage Analysis

Both cohorts were screened for coding and splice-site mutations in *C9orf72*. The frequency of rare mutations was determined in 400 controls. Further, we screened the Flanders-Belgian cohort for exonic deletions or duplications using the Multiplex Amplicon Quantification technique [Kumps et al., 2010]. For technical details see Supp. *Materials and Methods*.

### Genetic Association Studies

We calculated association with disease of *C9orf72* intermediate G_4_C_2_ alleles and of SNP rs2814707 tagging the chromosome 9p21 risk haplotype stratifying for the presence or absence of *C9orf72* intermediate repeats. Odds ratios (OR) with 95% confidence interval (CI) were calculated in a logistic regression model, adjusted for age and gender. In addition, we studied the correlation between the minor risk T-allele and intermediate repeat length. Further, in patients of both cohorts without a G_4_C_2_ expansion, we calculated correlation between age at onset and normal repeat length using a Kruskal–Wallis test. Further, we compared age at onset between short (2–6) and intermediate (7–24) repeat length in a Kaplan–Meier survival analysis. All analyses were done in IBM SPSS Statistics 20 (IBM Corporation, Armonk, NY, USA). For details see the Supp. *Materials and Methods*.

### Luciferase Reporter Assays

We selected a 2 kb *C9orf72* promoter fragment (chr9:27,572,414–27,574,451; NCBIBuild37 – hg19) containing the G_4_C_2_ repeat and enriched for histone marks, DNaseI hypersensitivity clusters, and transcription factor binding sites based on ENCODE transcription data [Gijselinck et al., 2012]. The fragment was obtained by PCR of individuals carrying different numbers of normal repeat units (2, 9, 17, and 24 units) using primers with flanked attB-sites. PCR products were cloned into the pDONR221 vector (Invitrogen, Life Technologies, Grand Island, NY, USA) by a BP recombination reaction (Invitrogen, Life Technologies, Grand Island, NY, USA), and the integrity of all inserts was confirmed by sequence analysis. Correct entry clones were selected and cloned into an in-house developed promoterless destination vector containing the *Gaussia* luciferase reporter gene downstream of a Gateway cassette, by use of a LR recombination reaction (Invitrogen, Life Technologies, Grand Island, NY, USA). Human HEK293T cells were propagated and seeded for transient transfection in 24-well tissue-culture dishes, at 2 × 10^5^ cells per well, and were allowed to recover for 24 hr. Cells were cotransfected with 40 ng of pSV40-CLuc plasmid that encodes the *Cypridina* luciferase gene with a SV40 promoter (New England Biolabs, Ipswich, MA, USA) and 1,000 ng of three independent *C9orf72* promoter constructs per unit, with use of 2.4 μl Lipofectamine 2000 (Invitrogen, Life Technologies, Grand Island, NY, USA), in duplo. After 24 hr, *Gaussia* luciferase activities (LA_G_) and *Cypridina* luciferase activities (LA_C_) were measured in duplo in the growth medium using a BioLux *Gaussia* and *Cypridina* Luciferase Assay Kit (New England Biolabs, Ipswich, MA, USA) and a Veritas Microplate Luminometer with Dual Reagent Injectors Luminometer (Promega, Madison, WI, USA). To correct for transfection efficiency and DNA uptake, the relative luciferase activity (RLA) was calculated as RLA = LA_G_/LA_C_. This experiment was repeated three times resulting in 36 measurements for each construct. RLAs between different repeat lengths were calculated by a Mann–Whitney *U* test. For details see Supp. *Materials and Methods*.

## Results

### The European EOD Consortium

The European cohort included 917 unrelated patients, of which 845 had a possible or probable diagnosis of FTLD (*n* = 781) or FTLD–ALS (*n* = 64; Table 1). In an additional 72 other patients, clinical presentation showed indications of FTLD together with symptomatology of other neurodegenerative brain diseases such as Alzheimer or Parkinson disease. A pathological diagnosis on autopsied brain was obtained for 45 patients, comprising FTLD-TDP (*n* = 28), FTLD-MND-TDP (*n* = 15), FTLD-UPS (*n* = 1), and FTLD-TAU (*n* = 1) diagnoses. Information on family history of disease was available for 609 (72.07%) of the 845 patients, of which 251 had a positive family history of disease and 358 were considered sporadic patients (Table 2). Average onset age and range were comparable between FTLD (62.7 ± 9.0, range 28–88 years) and FTLD–ALS patient groups (60.9 ± 9.8, range 31–83 years).

**Table 1:** Descriptive Characteristics of the European and Flanders-Belgian Cohorts

Clinical diagnosis cohorts	Total *n*	Familial *n* (%)	Disease onset ± SD (range)	Pathology *n* (%)
**European cohort**				
Total	845	274 (32.43)	62.5 ± 9.0 (28–88)	45 (5.33)
FTLD	781	251 (32.14)	62.7 ± 9.0 (28–88)	28 (3.59)
FTLD–ALS	64	23 (35.94)	60.9 ± 9.8 (31–83)	17 (26.56)
Other^a^	72	40 (55.56)	60.0 ± 12.1 (35–95)	N.A.
**Flanders-Belgian cohort**				
Total	360	108 (30.00)	62.9 ± 9.7 (29–85)	24 (6.67)
FTLD	337	101 (29.97)	63.0 ± 9.7 (29–85)	21 (6.23)
FTLD–ALS	23	7 (30.43)	62.7 ± 10.0 (39–75)	3 (13.04)
**Combined European and Flanders-Belgian cohorts**				
Total	1205	382 (31.70)	62.7 ± 9.2 (28–88)	70 (5.81)
FTLD	1118	352 (31.48)	62.8 ± 9.2 (28–88)	49 (4.38)
FTLD–ALS	87	30 (34.48)	61.4 ± 9.8 (31–83)	21 (24.14)

a This group of other 72 patients was not included in the total because they did not fulfill the criteria for possible or probable diagnosis.

**Table 2:** Frequencies of the *C9orf72* Pathological G_4_C_2_ Expansion in the European and Flanders-Belgian Cohorts

	FTLD		FTLD–ALS		Total	
**European cohort**						
Total	50/781	6.40%	23/64	35.94%	73/845	8.64%
Familial	21/251	8.37%	11/23	47.83%	32/274	11.68%
Sporadic	13/358	3.63%	5/27	18.52%	18/385	4.68%
Other^a^	16/172	9.30%	7/14	50.00%	23/186	12.37%
**Flanders-Belgian cohort**						
Total	21/337	6.23%	7/23	30.43%	28/360	7.78%
Familial	12/101	11.88%	6/7	85.71%	18/108	16.67%
Sporadic	9/236	3.81%	1/16	6.25%	10/252	3.97%
**Combined European and Flanders-Belgian cohorts**						
Total	71/1118	6.35%	30/87	34.48%	101/1205	8.38%
Familial	33/352	9.38%	17/30	56.67%	50/382	13.09%
Sporadic	22/594	3.70%	6/43	13.95%	28/637	4.40%
Other^a^	16/172	9.30%	7/14	50.00%	23/186	12.37%

a Other includes patients of which there was no family history data available in the European cohort.

### *C9orf72* Pathological G_4_C_2_ Expansions

To determine the impact and distribution of the G_4_C_2_ expansion across Europe, G_4_C_2_ repeat lengths were determined by forward RP-PCR (Fig. 1). A G_4_C_2_ expansion was observed in 8.64% (73/845) of the European cohort (Table 2). Per clinical subgroup, 6.40% of FTLD and 35.94% of FTLD–ALS patients carried a pathological G_4_C_2_ expansion. In familial patients, the overall frequency increased to 11.68%, with frequencies of 8.37% (21/251) in FTLD, and 47.83% (11/23) in FTLD–ALS patients. In sporadic patients, the overall frequency decreased to 4.68%, with frequencies of 3.63% (13/358) in FTLD, and 18.52% (5/27) in FTLD–ALS.

To evaluate the distribution of the G_4_C_2_ expansion, we calculated, overall and per clinical phenotype, mutation frequencies per country (Supp. Table S2; Table 3). In Belgium, Portugal, and Italy, the pathological G_4_C_2_ expansion mutation showed a comparable overall frequency ranging between 6.09% and 7.86%, and close to the average overall European frequency of 8.38%. However, a marked enrichment was observed in the Spanish (25.49%) and Swedish (21.33%) patient cohorts. In contrast, in the German patients, only 3.52% were carriers.

**Table 3:** Meta-analysis of *C9orf72* Prevalence Studies in Western Europe

	Total			Familial			Sporadic			
				
Country	Total *n*	Carriers *n*	Carriers (%)	Total *n*	Carriers *n*	Carriers (%)	Total *n*	Carriers *n*	Carriers (%)	Studies represented in the meta-analysis
Total^a^	2636	263	9.98	756	140	18.52	1804	113	6.26	
Belgium	369	29	7.86	115	19	16.52	254	10	3.94	Gijselinck et al. (2012), European EOD consortium
Denmark	82	10	12.20	N.A.	N.A.	N.A.	N.A.	N.A.	N.A.	Lindquist et al. (2012)
Finland	75	22	29.33	27	13	48.15	48	9	18.75	Majounie et al. (2012)
France	200	36	18.00	50	22.00	44	150	14	9.33	Majounie et al. (2012)
Germany	228	11	4.82	66	5	7.58	162	6	3.7	European EOD consortium, Majounie et al. (2012)
Italy	345	21	6.09	86	5	5.81	259	16	6.18	European EOD consortium
The Netherlands	340	35	10.29	116	30	25.86	224	5	2.23	Majounie et al. (2012)
Portugal	151	10	6.62	92	4	4.35	59	6	10.17	European EOD consortium
Spain	51	13	25.49	20	6	30	31	7	22.58	European EOD consortium
Sweden	82	17	20.73	14	8	57.14	74	9	12.16	European EOD consortium, Majounie et al. (2012)
UK	713	59	8.27	170	28	16.47	543	31	5.71	Majounie et al. (2012)

Meta-analysis combined prevalences from the European EOD consortium study, the Gijselinck et al., Lancet Neurol 2012 study [Gijselinck et al., 2012], the Majounie et al., Lancet Neurol 2012 study [Majounie et al., 2012], and the Lindquist et al., Clin Neurol study [Lindquist et al., 2012].

a Patient samples from the Czech Republic, Bulgaria, and Austria from the European EOD consortium study, and from Sardinia from the Majounie study, contained less than 20 patients and were therefore excluded from the meta-analysis. N.A.: information not available.

### *C9orf72*-Associated Clinical and Pathological Phenotype

Of 73 G_4_C_2_ expansion carriers in the European cohort, 50 received a clinical diagnosis of FTLD and 23 of FTLD–ALS. The average onset age in all carriers was 58.0 ± 7.5 years with an onset age range of 40–75 years and was comparable in the FTLD and FTLD–ALS subgroups (Supp. Table S3). The average disease duration in 31 deceased carriers was 5.3 ± 3.5 years (range 1–14 years) but differed in the clinical subgroups. Survival was on average 1.4 years shorter for the FTLD–ALS carriers with 4.7 ± 3.5 years (*n* = 17, range 1–14 years), compared with the FTLD patients with 6.1 ± 3.6 years (*n* = 14, range 1–14 years). Of 23 G_4_C_2_ expansion carriers, more extensive clinical information was available to allow subclassification to the different FTLD phenotypes. In 22, clinical presentation was conform bvFTD (95.65%) and one presented with PNFA. Autopsied brain was available for 11 European G_4_C_2_ expansion carriers (two Portuguese, five Spanish, one Austrian, one Czech, and two Swedish patients). In all 11 cases, we found TDP-43 pathology in the frontal and temporal neocortex, in the hippocampus and neostriatum, which was compatible with type B TDP proteinopathy [Mackenzie et al., 2010]. Accordingly, TDP-43 immunoreactive neuronal cytoplasmic inclusions (NCI) and dystrophic neurites were widespread over the entire cortical thickness, but NCI were more pronounced in the pyramidal cells of layer 2 compared with the deeper cortical layers. In addition to the TDP-43 positive pathology, p62 immunoreactive NCI were present in the granular layer of the dentate gyrus of the hippocampus, and in the granular layer of the cerebellar cortex. Further, p62 positive irregular granular NCI were observed in pyramidal neurons of the CA4 and CA3 region of the hippocampus.

### Genomic Complexity in the *C9orf72* Region

The *C9orf72* G_4_C_2_ repeat is contiguous with a GC-rich, LCS, comprising exon 1 of the *C9orf72* transcript NM_018325.3 (Fig. 1). We sequenced the GC-rich LCS in 317 unrelated patients of the Flanders-Belgian cohort and in 752 controls (Fig. 1). We observed heterozygous deletions of 5–23 base pairs (bp) in a total of 19 individuals of which 10 patients carrying a G_4_C_2_ expansion (10/27 = 37.04%), five noncarrier patients (5/290 = 1.72%), and four control persons (4/752 = 0.53%; Table 4). These variable deletions were significantly more frequently observed in carriers of a G_4_C_2_ expansion compared with the group of noncarrier patients (OR = 33.53; 95% CI 10.31–109.09; *P* < 0.001) and controls (OR = 93.50; 95% CI 28.53–306.39; *P* < 0.001). Remarkably, nine of 10 (90.00%) expansion carriers presented with the same heterozygous 10-bp GTGGTCGGGG deletion (g.26747_26756 del**GTGGT**CGGGG; Supp. Fig. S1), which was not observed in patient noncarriers and controls (Table 4). This 10-bp deletion is contiguous with the G_4_C_2_ repeat and joins two 100% GC sequences, thereby extending the GC-rich motif of the G_4_C_2_ repeat with imperfect repeats (Fig. 1). In this context, it is striking that deletion of the GTGGT motif (Fig. 1) was seen in all 10 deletion carriers of the 27 unrelated patients carrying an expanded G_4_C_2_ repeat (37.04%), only once in the noncarrier patients (1/290 = 0.34%) and once in control individuals (1/752 = 0.13%; Table 4). To replicate these findings, we successfully sequenced the LCS in 57 unrelated patient carriers and 114 patient noncarriers from the European cohort. We observed a comparable high frequency of deletions and insertions (indels) in the patient carriers (14/57 = 24.56%) and no indels in the noncarriers (0/114, <0.88%; OR = 36.79; 95% CI 4.69–288.35; *P* = 0.001; Table 4). Seven of 14 (50.00%) patient carriers presented the same 10-bp deletion and in 10 of 14 (71.43%) patient carriers GTGGT was deleted (Table 4). Of note, children of the FTLD patient with the g.26747_26751del**GTGGT** deletion but without expansion did not show de novo expansions. This deletion is located on an allele of 5 repeat units.

**Table 4:** Low Complexity Sequence (LCS) Indels in Carriers of a Pathological G_4_C_2_ Expansion and in Noncarriers of the Flanders-Belgian and European cohorts

Diagnosis	Number of individuals	Indel genomic mutation name^a^,^b^
**Flanders-Belgian cohort**		
**Patients expansion carriers (10/27)**		
FTLD	7	g.26747_26756del**GTGGT**CGGGG
FTLD–ALS	2	g.26747_26756del**GTGGT**CGGGG
FTLD–ALS	1	g.26742_26764delGGGGC**GTGGT**CGGGGCGGGCCCG
**Patients without expansion (5/290)**		
FTLD	1	g.26747_26751del**GTGGT**
FTLD	3	g.26752_26762delCGGGGCGGGCC
FTLD—ALS	1	g.26752_26774delCGGGGCGGGCCCGGGGGCGGGCC
**Controls (4/752)**		
Control	3	g.26752_26762delCGGGGCGGGCC
Control	1	g.26746_26761delC**GTGGT**CGGGGCGGGC
**European cohort**		
**Patients expansion carriers (14/57)**		
FTLD	6	g.26747_26756del**GTGGT**CGGGG
FTLD	1	g.26747_26751del**GTGGT**
FTLD	1	g.26747_26768del**GTGGT**CGGGGCGGGCCCGGGGG
FTLD	1	g.26753_26764delGGGGCGGGCCCG
FTLD	1	g.26775_26776insG
FTLD	1	g.26775_26776insGGGGCGGGCCCG
FTLD–ALS	1	g.26747_26756del**GTGGT**CGGGG
FTLD–ALS	1	g.26746_26773del C**GTGGT**CGGGGCGGGCCCGGGGGCGGGC
FTLD–ALS	1	g.26752_26762delCGGGGCGGGCC

a The nucleotide sequence GTGGT is most frequently deleted in the LCS adjacent to the G_4_C_2_ repeat in *C9orf72* (indicated in bold).

b gDNA numbering relative to reverse complement of contig AL451123.12 and starting at nucleotide 1.

The LCS is comprised in the PCR fragments produced by the forward RP-PCR assay to identify G_4_C_2_ expansion carriers (Fig. 1) [Gijselinck et al., 2012]. To eliminate influences of LCS variability in G_4_C_2_ expansion detection and for sizing normal repeat alleles, we developed a reverse RP-PCR assay on the sense strand (Fig. 1). This assay confirmed the presence of the G_4_C_2_ expansion in patients of both cohorts.

### Sizing of Normal *C9orf72* G_4_C_2_ Repeat Lengths

**Figure 2:**
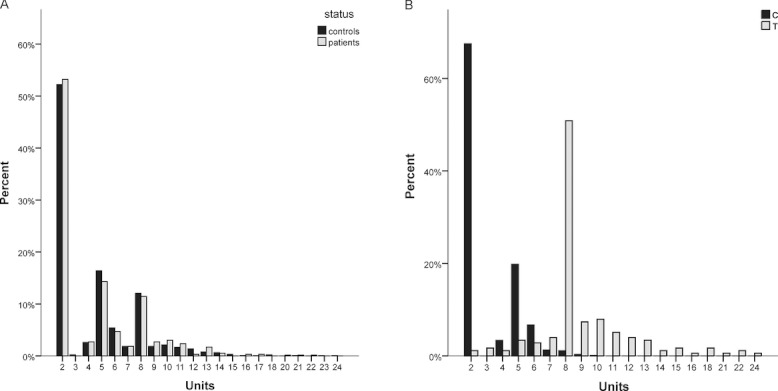
**A**: Distribution of normal repeat lengths in the Flanders-Belgian patients and control individuals. Histograms of G_4_C_2_ repeat units sized <60 repeats in Flanders-Belgian patients, excluding patients with mutations in known causal genes or with a pathological G_4_C_2_ expansion, compared with control individuals. **B**: Correlation of normal repeat lengths with rs2814707 alleles. Histograms of G_4_C_2_ repeat units in 610 control individuals homozygous for the rs2814707 C-allele and 53 homozygous for the rs2814707 T-allele.

We used a STR genotyping assay (STR-PCR; Fig. 1), to size both normal alleles in noncarriers and the wild-type allele in G_4_C_2_ expansion carriers. In addition, we used the reverse RP-PCR assay (Fig. 1) to validate the size of the longest allele in nonexpansion carriers. When we compared the allele lengths between the two assays, we obtained 99% concordance. Discordant allele scoring could be explained by the presence of a LCS deletion. This implies that the STR-PCR can be used for correct sizing of normal repeat alleles. The observed lengths of the G_4_C_2_ repeat ranged from 2 to 24 units in nonexpansion carriers of the Flanders-Belgian cohort (g.26724GGGGCC[2_24]; relative to reverse complement of AL451123.12; Fig. 2A) and between 2 and 21 units in the European cohort. The shortest allele contained one unit less than the reference genome (NCBIbuild37–hg19; Fig. 1). Frequency distribution of normal repeat alleles showed a trimodal allele distribution (Fig. 2A). Using an estimation–maximization algorithm implemented in the R Package MCLUST, the three groups could be defined by a cutoff at 4 and 7 units (2–3 units, 4–6 units, and 7–24 units), with a probability of good classification of 0.9944.

### Intermediate Repeat Length and the Chromosome 9 Risk Haplotype

In the Flanders-Belgian cohort, we previously had calculated a significant association of disease with the risk T-allele of rs2814707 (*P* = 0.008) [Gijselinck et al., 2012] tagging the chromosome 9p risk haplotype. Repeat expansion carriers contributed the largest fraction of the attributed risk but residual association signal remained in the nonexpansion carriers homozygous for the T-allele (OR = 1.75; 95% CI 1.02–3.01; *P* = 0.042; Supp. Table S4) [Gijselinck et al., 2012]. We used three different PCR genotyping assays to test for pathological G_4_C_2_ expansions, making it unlikely that the residual association could be explained by missed mutation carriers. Also, we analyzed *C9orf72* for other mutations by sequencing all exons in 493 patients and by exon-based analysis of putative deletions/duplications in 413 patients. Except for one patient-specific missense mutations (c.196A>T in NM_018325.3 [p.Thr66Ser]) without a clear in silico deleterious effect, no other pathogenic mutations were identified. When we compared the distribution of normal repeat lengths between the rare T-allele and the common C-allele, we observed that alleles of at least 7 repeat units were strongly correlated with the T-allele (*P* < 0.001; Fig. 2B), corresponding with allele group 3 of the trimodal frequency distribution. We defined this group (7–24 units) as intermediate repeat alleles. When we recalculated genetic association of rs2814707 with disease after excluding individuals homozygous for intermediate alleles, the residual association disappeared (*P* = 0.121; Supp. Table S4). However, we could not demonstrate direct association of these intermediate alleles with disease (Supp. Table S5). Although a slight increase in frequency of intermediate alleles (25.1%) was observed as compared with the control individuals (22.6%; Supp. Table S5). Also homozygous intermediate repeat carriers were slightly more frequent in the patient group (6.1% vs. 4.6%).

We could not observe evidence in favor of the hypothesis that intermediate repeats are unstable and might trigger pathological G_4_C_2_ expansion, as the younger generation in 20 families of index patients with an intermediate repeat allele up to 22 units, did not show longer intermediate repeat alleles or pathological expansions.

### Reporter Gene Analysis

**Figure 3:**
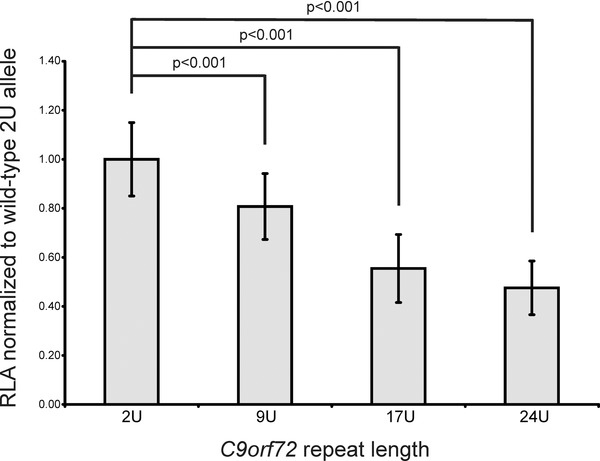
Transcriptional activity of *C9orf72* promoter with alleles of different repeat length. Bars represent relative *Gaussia*/*Cypridina* luciferase activities (RLA) for the different *C9orf72* constructs compared with the wild-type allele of 2 units, for an increasing amount of repeat units. Values represent the mean (±SDEV) of 36 independent measurements relative to the 2 units wild-type allele. The significance of differences in expression was calculated using the Mann–Whitney *U* test. *P* values are presented above the bars.

To evaluate the effect of repeat length on *C9orf72* promoter activity, we measured the RLA of constructs containing a *C9orf72* promoter fragment with 2, 9, 17, and 24 units. We demonstrated a highly significant decrease of the RLA between fragments with 9, 17, and 24 units compared with the wild-type allele comprising 2 units (*P* < 0.001) with a maximum decrease of 52% in the 24 units containing promoter (Fig. 3). These data show that intermediate repeat alleles result in a significantly reduced *C9orf72* promoter activity compared with the wild-type alleles.

## Discussion

In the present study, we have assessed the geographical distribution of the pathological G_4_C_2_ expansions in an extended pan-European patient cohort of FTLD patients, originating from Italy, Germany, Portugal, Sweden, Spain, Czech Republic, Bulgaria, Austria, and Belgium, ascertained within a newly formed, European EOD consortium. The prevalence of pathological G_4_C_2_ expansions in the total European cohort (73/845, 8.64%) as well as in the FTLD-only (50/781, 6.40%) and FTLD–ALS (23/64, 35.94%) subgroups, was comparable to frequencies others and we published in the three original gene identification reports on *C9orf72* [Dejesus-Hernandez et al., 2011; Gijselinck et al., 2012; Renton et al., 2011].

The FTLD syndrome in the European G_4_C_2_ expansion carriers was most often characterized by behavioral disturbances (95.7%), which was in line with our observations from an in-depth genotype–phenotype correlation study in the Belgian *C9orf72* carriers [Van Langenhove et al., 2012b]. Thirty-two percent of expansion carriers also developed concomitant ALS symptomatology. Further, we observed a wide range in onset age and disease duration, suggestive of genetic and/or environmental (epigenetic) disease modifiers [Van Langenhove et al., 2012b]. Of 13 G_4_C_2_ expansion carriers, post-mortem neuropathological diagnosis confirmed brain deposition of TDP-43 inclusions, explaining 30.23% (13/43) of TDP-43 positive patients. Because up to 70% of the TDP pathology patients remained unresolved after *C9orf72* analysis, we performed mutation profiling of the other TDP-43-associated genes *GRN*, *VCP*, and *TARDBP*. This revealed one additional *TARDBP* mutation, p.Ile383Val, in a patient from the Barcelona Brain Bank. Interestingly, this mutation located in the C-terminal of the protein was reported once before in one US ALS family [Rutherford et al., 2008], yet in this Spanish patient—diagnosis SD, age at onset 60 years, age at death 75 years, father with dementia—no clinical nor pathological signs of MND were recorded. Taken together, of the 43 TDP-43 pathology patients 67.44% (29/43) remained unresolved by the known genes. In 17 familial TDP patients, this represented 58.82% (10/17) of patients without a known mutation, pointing to at least one, but likely more, other TDP-43-associated genes yet to be discovered. Eleven *C9orf72* positive TDP-43 pathology carriers were submitted to further in-depth histopathological evaluation. Although the neuropathological signature of *C9orf72* has proven to be more diverse than initially anticipated (reports ranged from strong TDP proteinopathy reminiscent of FTLD-TDP type A [Murray et al., 2011; Snowden et al., 2012; Stewart et al., 2012], to complete absence of TDP-43 pathology [Gijselinck et al., 2012; Murray et al., 2011] (reviewed in Cruts et al., Trends Neurosci, under revision), patients in our study all fitted the criteria of TDP proteinopathy type B [Mackenzie et al., 2010], albeit with relatively low lesion load. Eight of the 11 patients displayed concomitant AD-pathology, yet, AD stages were relatively mild (Braak stage I–III for neurofibrillary tangle pathology and Stage B for β-amyloid pathology) and probably this finding was age related more than related to the *C9orf72* expansion. In the five cases stained with p62 antibody, 100% showed immunoreactive NCI in the dentate gyrus of the hippocampus, in the CA4 and CA23 pyramidal neurons of the hippocampus, and the granular cortical layer of the cerebellum. As this lesion load is far more pronounced than the TDP-43 lesions, it can be assumed that the presence of p62 pathology is a highly distinctive feature of *C9orf72*-associated FTLD [Al-Sarraj et al., 2011]. In another recent large-scale screening of the *C9orf72* repeat expansion by Majounie et al. (2012), frequencies were reported for a total of 4,448 ALS patients and 1,425 FTLD patients from European and US Caucasian populations, and compared with smaller sets of patients from other ethnic backgrounds. Of interest with respect to the present study, the Majounie study included FTLD patients from the UK, the Netherlands, France, Finland, Germany, Sardinia, and Sweden. Except for a small number of German (*n* = 29) and Swedish (*n* = 7) patients, these countries were not represented in our study and vice versa. Further, a study conducted in Denmark identified 10 *C9orf72* expansion carriers in a cohort of 82 Danish FTLD patients [Lindquist et al., 2012]. To get an even more representative picture of the prevalence and distribution of the *C9orf72* repeat expansion in Western Europe, we performed a meta-analysis of the FTLD cohorts from our European EOD consortium study (*n* = 845), our previously reported Flanders-Belgian cohort (*n* = 360) [Gijselinck et al., 2012], the Majounie study (*n* = 1381) [Majounie et al., 2012], and the Lindquist study (*n* = 82) [Lindquist et al., 2012]. This resulted in prevalence numbers on 2,668 FTLD patients from 15 European countries (Table 3, countries with patients groups smaller than 20 were not included in the meta-analysis). The overall frequency of the *C9orf72* pathological G_4_C_2_ expansion in Western Europe was 9.98% in total FTLD, 18.52% in familial FTLD, and 6.26% in sporadic FTLD. Looking at the per country distribution, Belgium, Italy, The Netherlands, Portugal, and the UK showed an average total prevalence ranging from 6.09% to 10.29%. Denmark showed a slightly increased prevalence of 12.20%, and in France the prevalence was about double the average prevalence with 18.00%. In line with the hypothesis of a Scandinavian founder effect for the chromosome 9—*C9orf72* haplotype [Mok et al., 2011], frequencies were highest in Finland (29.33%) and high in Sweden (20.73%). Yet, in contrast with this hypothesis and the expected North–South axis for the founder haplotype prevalence, frequencies for *C9orf72* reached 25.49% in Spain. To the other extreme, prevalence in Germany was just 4.82%. Of note, Ireland, Luxembourg, Switzerland, Poland, Norway, and Greece are not yet represented in this meta-analysis of Western Europe.

We exactly sized the normal range of repeat alleles in both the Flanders-Belgian and European cohorts using two different assays and observed a range between 2 and 24 units. The allele frequencies showed a trimodal distribution with a long tail containing the rare longer alleles. Alleles in group 3 of this trimodal distribution (intermediate alleles; 7–24 units) are almost solely present on the chromosome 9 risk haplotype tagged by the rs2814707 T-allele. We asked the question whether these alleles might act as a risk factor on FTLD disease susceptibility or could have a mild effect on gene expression. Although intermediate repeat alleles were not significantly associated with disease in the Flanders-Belgian cohort, the slight increase in frequency in patients compared with control individuals explained the residual association we had observed for homozygous carriers of the rs2814707 T-allele (OR = 1.75; 95% CI 1.02–3.01; *P* = 0.042). A group of 16 FTLD–ALS patients without pathological expansion was included in this cohort. Although this is a small group, it is remarkable that seven of 32 alleles (21.9%) have at least 10 units, which is significantly higher than 7.2% in control individuals (OR = 3.685; 95% CI 1.560–8.706; *P* = 0.003). This suggests that intermediate alleles might rather have a risk effect in diseases involving ALS. Because the European cohort was collected for a frequency study of the repeat expansion, we did not have ethnicity matched control individuals for an association study.

Flanders-Belgian and European patient carriers of intermediate repeats (7–24) did not show an earlier onset age compared with carriers of shorter repeats (2–6; data not shown). Also, the variable onset age in patients with a pathological G_4_C_2_ expansion [Gijselinck et al., 2012], could not be explained by a modifying effect of number of repeat units in the wild-type allele (data not shown), in contrast to what has been shown for patients with Huntington's disease [Aziz et al., 2009].

The mechanism by which the G_4_C_2_ intermediate repeats might contribute to disease pathogenesis remained elusive. In this study, we performed reporter gene expression studies to evaluate the effect of intermediate repeats according to their size on *C9orf72* promoter activity. We provided compelling evidence that transcriptional activity of the *C9orf72* promoter significantly decreases with an increasing amount of repeat units with a maximal reduction of 52% of a 24 units containing promoter compared with 2 units on the wild-type allele (Fig. 3). To better discriminate the cutoff from which decreased transcriptional activity is apparent, more different length alleles should be investigated, although from these data it is clear that intermediate alleles affect the normal transcriptional activity of the *C9orf72* promoter. With the cloned intermediate alleles, we did not reach an expression level of virtually 0 as expected in pathogenic expansion alleles. Therefore, individuals with the number of units higher than 24 are needed to better interpret the grey zone between normal and expanded alleles. These in vitro data confirm that the G_4_C_2_ repeat is located in the promoter region of *C9orf72* as we previously suggested [Gijselinck et al., 2012]. It also favors the loss-of-function hypothesis that was based on 50% reduction of *C9orf72* levels in brain of pathological expansion carriers preventing expression of the mutant allele [Gijselinck et al., 2012]. However, in this study, we did not identify other simple sequence mutations or deletions/duplications pointing to a loss-of-function mechanism, except for one patient-specific missense mutation (p.Thr66Ser) without clear pathogenic nature. Therefore, other molecular mechanisms might also contribute to disease including sequestration of RNA-binding proteins and RNA toxicity [Dejesus-Hernandez et al., 2011], which can also lead to a decrease of the mRNA level. These two potential disease mechanisms are not mutually exclusive, and might also lead together to degeneration of neuronal populations in either the frontal cortex (FTLD) or the spinal cord (ALS).

By what mechanisms the G_4_C_2_ repeat expands to a pathological size range remains to be discovered. In our first *C9orf72* study in the Flanders-Belgian cohort [Gijselinck et al., 2012], we showed that the majority of the pathological G_4_C_2_ expansions were located on the same risk haplotype tagged by the rare T-allele of SNP rs2814707, and strongly associated with FTLD and ALS. These observations were confirmed in the European study with 100% of European carriers of a pathological G_4_C_2_ expansion carrying at least one T-allele. It has been proposed that this tight genetic association might be explained by a single founder mutation on this risk haplotype [Mok et al., 2011; Majounie et al., 2012]. An alternative hypothesis is that a specific genomic context on this risk haplotype is rendering the G_4_C_2_ repeat less stable and making it more prone to expansion into the pathological size range. In this context, we and others [Dejesus-Hernandez et al., 2011] showed that the G_4_C_2_ intermediate repeats are also highly significantly overrepresented on the same risk haplotype, suggesting that these intermediate repeats are more prone to replication slippage and unstable inheritance and thus triggering pathological expansions. The observation that intermediate alleles have the same linkage disequilibrium with a specific SNP allele as the expanded alleles is also previously made in other repeat expansion disorders, for example, Fragile X Syndrome [Gunter et al., 1998]. The appearance of pathological G_4_C_2_ expansions in apparently sporadic patients in this and previous studies [Dejesus-Hernandez et al., 2011; Gijselinck et al., 2012; Renton et al., 2011], seems to support this hypothesis. However, we could not observe increasing G_4_C_2_ repeat length or de novo expansions in the younger generations of families of index patients with intermediate repeat length up to 22 units. Therefore, intermediate alleles might rather be considered as predisposing alleles for further stepwise expansion over probably many generations instead of pre-mutations. A study using sporadic ALS trios did also not show evidence of repeat instability between two generations [Pamphlett et al., 2012].

Further, we observed in patients of both the Flanders-Belgian and European cohorts with a pathological G_4_C_2_ expansion, a significantly higher frequency of short indels in the GC-rich LCS adjacent to the G_4_C_2_ repeat (24/84, 28.57%) compared with patient noncarriers (5/404, 1.24%; *P* < 0.001) and control persons (4/752, 0.53%; *P* < 0.001). Remarkably, in 23.81% (20/84) of repeat expansion carriers, a deletion of the GTGGT motif contiguous with the G_4_C_2_ repeat was comprised within the indel (Fig. 1), compared with 0.25% in the noncarrier patients and 0.13% in control individuals (Table 1). This GTGGT deletion joins the two GC-rich sequences, increasing the overall GC content of the LCS. This has already been reported for CAG expansion diseases, where the expandability of the repeat increases with a higher GC-content of the surrounding DNA sequence [Brock et al., 1999; Nestor and Monckton, 2011]. Also, the GTGGT deletion creates one long contiguous imperfect G_4_C_2_ repeat, which is likely more prone to replication slippage. Therefore, the GTGGT sequence is potentially an important stabilizer of the G_4_C_2_ repeat. This resembles the loss of AGG interspersion in unstable Fragile X alleles [Kunst and Warren, 1994; Larsen et al., 2000]. Moreover, all carriers of a GTGGT deletion in the LCS had at least one rs2814707 T-allele and the 10-bp deletion was cotransmitted with the G_4_C_2_ expansion in relatives of two expansion carriers, which indicates that it is likely located on the risk haplotype and might trigger the expansion. Nevertheless, it should be noted that not all carriers of a pathological G_4_C_2_ expansion also have an LCS indel and thus we cannot exclude that the indels are rather a consequence of the close neighborhood of the expansion destabilizing the genomic region. Of note, the indels found in nonexpansion carriers are mostly not affecting the GTGGT sequence but rather stabilizing the repeat by deleting 100% GC-rich sequence. The GTGGT deletion in one FTLD patient without repeat expansion is located on a short allele with 5 units, which is probably not unstable enough to expand. A GTGGT deletion on an intermediate allele will most likely be not stable and hence not observed.

A better understanding of the underlying mechanism causing the G_4_C_2_ instability will be essential to assess disease risk and improve clinical benefits. Further, elucidating how G_4_C_2_ expansions lead to disease will be crucial to unveil biological pathways and key molecules in the disease process as targets for future therapies.

## Appendix

European EOD Consortium members that contributed to the data in this article:

*Department of Molecular Genetics, VIB, Antwerp, Belgium, Neurodegenerative Brain Diseases Group*: Julie van der Zee, Ilse Gijselinck, Lubina Dillen, Tim Van Langenhove, Jessie Theuns, Stéphanie Philtjens, Kristel Sleegers, Veerle Bäumer, Githa Maes, Ellen Corsmit, Marc Cruts, Christine Van Broeckhoven; *Neurogenomics Group*: Albena Jordanova

*Institute Born-Bunge, University of Antwerp, Antwerp, Belgium, Laboratory of Neurogenetics*: Julie van der Zee, Ilse Gijselinck, Lubina Dillen, Tim Van Langenhove, Stéphanie Philtjens, Jessie Theuns, Kristel Sleegers, Veerle Bäumer, Githa Maes, Marc Cruts, Christine Van Broeckhoven; *Laboratory of Neurochemistry and Behavior*: Sebastiaan Engelborghs, Peter P. De Deyn; *Laboratory of Neurobiology*: Patrick Cras

*Department of Neurology and Memory Clinic, Hospital Network Antwerp, Middelheim and Hoge Beuken, Antwerp, Belgium*: Sebastiaan Engelborghs, Peter P. De Deyn

*University of Leuven, Leuven, Belgium, Brain and Emotion Laboratory*: Mathieu Vandenbulcke; Laboratory for Cognitive Neurology: Rik Vandenberghe

*University Hospitals Leuven Gasthuisberg, Leuven, Belgium, Department of Psychiatry*: Mathieu Vandenbulcke; *Department of Neurology*: Rik Vandenberghe

*Centre for Ageing Brain and Neurodegenerative Disorders, Neurology Unit, University of Brescia, Brescia, Italy*: Barbara Borroni, Alessandro Padovani, Silvana Archetti

*Department of Psychiatry and Psychotherapy, Technische Universität München, Munich, Germany*: Robert Perneczky, Janine Diehl-Schmid

*Department of Neurodegeneration, Hertie Institute for Clinical Brain Research and Centre of Neurology, Tuebingen, Germany*: Matthis Synofzik, Walter Maetzler, Jennifer Müller vom Hagen, Ludger Schöls

*German Research Center for Neurodegenerative Diseases (DZNE), University of Tuebingen, Germany*: Matthis Synofzik, Walter Maetzler, Jennifer Müller vom Hagen, Ludger Schöls; *University of Bonn, Germany*: Michael T. Heneka, Frank Jessen, Alfredo Ramirez, Delia Kurzwelly, Carmen Sachtleben, Wolfgang Mairer

*Institute of Molecular Medicine, Faculty of Medicine, University of Lisbon, Lisbon, Portugal*: Alexandre de Mendonça, Gabriel Miltenberger-Miltenyi, Sónia Pereira, Clara Firmo

*Faculty of Medicine, University of Lisbon, Lisbon, Portugal*: José Pimentel

*Alzheimer's disease and Other cognitive disorders unit, Department of Neurology, Hospital Clinic, Barcelona, Spain*: Raquel Sanchez-Valle, Albert Llado, Anna Antonell, Jose Molinuevo

*Biobanc, Hospital Clinic, Institut d'Investigacions Biomèdiques August Pi i Sunyer (IDIBAPS), Barcelona, Spain*: Ellen Gelpi

*KI'Alzheimer Disease Research Center, Karolinska Institutet, Stockholm, Sweden, Department of Neurobiology, Care Sciences and Society*: Caroline Graff, Huei-Hsin Chiang, Marie Westerlund; *Department of NVS*: Charlotte Forsell, Lena Lillius

*Karolinska University Hospital, Stockholm, Sweden, Department of Geriatric Medicine, Genetics unit*: Caroline Graff, Anne Kinhult Ståhlbom, Håkan Thonberg; *Department clinical cytology and pathology*: Inger Nennesmo

*Department of Psychiatry and Neurochemistry, Institute of Neuroscience and Physiology, Sahlgrenska Academy, University of Gothenburg*: Anne Börjesson-Hanson

*Department of Neurological and Psychiatric Sciences, University of Florence, Florence, Italy*: Benedetta Nacmias, Silvia Bagnoli, Sandro Sorbi, Valentina Bessi, Irene Piaceri

*Neurology Department, Centro Hospitalar Universitário de Coimbra, Coimbra, Portugal*: Isabel Santana, Beatriz Santiago

*University of Coimbra, Coimbra, Portugal, Faculty of Medicine*: Isabel Santana, Maria Helena Ribeiro; *Center for Neuroscience and Cell Biology*: Maria Rosário Almeida, Catarina Oliveira

*Department of Neurology Centro Hospitalar de São João, and Department of Clinical Neuroscience and Mental Health, Faculty of Medicine University of Porto, Porto, Portugal*: João Massano, Carolina Garret

*Hospital de Angra do Heroísmo, Azores, Portugal*: Paula Pires

*Laboratory of Epidemiology Neuroimaging & Telemedicine & National Centre for Alzheimer's and Mental Diseases, IRCCS Centro San Giovanni di Dio, FBF, Brescia, Italy*: Giovanni Frisoni, Orazio Zanetti, Cristian Bonvicini

*Medical University Sofia, Sofia, Bulgaria, Department of Neurology*: Stayko Sarafov, Ivailo Tournev; Molecular Medicine Center, Department of Biochemistry: Albena Jordanova

*Department of Cognitive Science and Psychology, New Bulgarian University, Sofia, Bulgaria*: Ivailo Tournev

*Institute of Neurology, Medical University Vienna, Vienna, Austria*: Gabor G. Kovacs, Thomas Ströbel

*University of Bonn, Germany, Department of Neurology, Clinical Neuroscience Unit*: Michael T. Heneka, Frank Jessen, Alfredo Ramirez, Delia Kurzwelly, Carmen Sachtleben, Wolfgang Mairer; *Department of Psychiatry*: Frank Jessen

*Department of Pathology and Molecular Medicine, Thomayer Hospital, Prague, Czech Republic*: Radoslav Matej, Eva Parobkova

*Ludwig-Maximilians-Universität Munich, Germany, Neurologische Klinik*: Adrian Danel; *Zentrum für Neuropathologie und Prionforschung*: Thomas Arzberger

*Section of Neuropathology, Department of Neurological, Neuropsychological, Morphological and Movement Sciences, University of Verona, Italy*: Gian Maria Fabrizi, Silvia Testi

*Unità Operativa Complessa di Neuropatologia d'U, Azienda Ospedaliera Universitaria Integrata di Verona*: Sergio Ferrari, Tiziana Cavallaro

*Cyclotron Research Centre and Department of Neurology, University of Liège, Liège, Belgium*: Eric Salmon

*Department of Neurology, University Hospital Ghent and University of Ghent, Ghent, Belgium*: Patrick Santens

*Department of Neurology, Antwerp University Hospital, Edegem, Belgium*: Patrick Cras

## Supplementary material

Disclaimer: Supplementary materials have been peer-reviewed but not copyedited.
