# Supplementary material for: A Pan-European Study of the C9orf72 Repeat Associated with FTLD: Geographic Prevalence, Genomic Instability, and Intermediate Repeats
Source: Hum Mutat. 2012 Oct 30;34(2):363–73. doi: 10.1002/humu.22244 (PMC3638346; doi:10.1002/humu.22244)
Supplement: Supplementary file 1 [file humu0034-0363-SD1.pdf]

SUPP. MATERIALS AND METHODS

Flanders-Belgian cohort

Index patients were evaluated using a standard clinical diagnostic protocol, including detailed recording of clinical and family history, neurological examination, neuropsychological testing, biochemical analyses and neuroimaging. Additional patients were included who had initially been referred to the DMG DSF for clinical genetic testing. Patients were diagnosed with behavioral variant FTD (bvFTD), progressive non-fluent aphasia (PNFA) or semantic dementia (SD) according to consensus diagnostic criteria for FTLD [Neary et al., 1998]or with concomitant FTLD-ALS. Postmortem analysis confirmed clinical diagnosis in 21 FTLD and 3 FTLD-ALS patients.

Mutation analyses of FTLD (*C9orF72*, *GRN*, *MAPT*, *VCP* and *CHMP2B*) and ALS (*C9orF72*, *TARDBP*, *FUS*, *ATXN2* and *VCP*) genes identified pathogenic mutations in 60 (16.7%) of the FTLD patients [Van Langenhove et al., 2012; Gijselinck et al., 2012]. Age-matched community controls were recruited from the same geographical region of Flanders-Belgian as the patients. All were submitted to a medical survey inquiring about personal as well as family medical history of neurodegenerative or psychiatric disease, followed by a Mini Mental State Examination test (MMSE). Only participants free of the aforementioned diseases and with a MMSE score > 26 were included in the control population (n=1083).

Ethical assurance

All participants or their legal guardian to the European and Belgian cohort gave written informed consent for participation in the clinical and genetic studies, and for brain autopsy if appropriate. The clinical study protocol and the informed consent forms for patient ascertainment were approved by the local Medical Ethics Committees of the collaborating centers. The genetic and pathological study protocols and informed consent forms were approved by the Medical Ethics Committee of the University Hospital of Antwerp, Belgium.

Histopathology of *C9orf72* expansion carriers

Post-mortem delay before fixation varied between 3 and 24 hours. After a fixation period of 4 to 8 weeks in 10% buffered formalin, 5µm slices were cut. Samples were obtained from frontal cortex, temporal neocortex, hippocampus, area striata, neostriatum, mesencephalon, pons and cerebellum. Of seven cases we had samples of thalamus and spinal cord. For histological analysis cresyl-violet, hematoxylin-eosin staining and Kluver-Barrera staining were used. Immunohistochemical analysis was performed with anti-ubiquitin antibody (Dako, Glostrup, Denmark), p62 antibody (p62 mAb BD Biosciences, Erembodegem Belgium), AT8 against hyperphosphorylated tau (Innogenetics, Zwijnaarde, Belgium), 4G8 against β-amyloid (Signet, Dedham, Massachusetts), anti-FUS antibody (Sigma Aldrich, St Louis), anti-TDP-43 antibody (Proteintech Group Inc, Chicago, Illinois).

C9orf72 G_4C_2 genetic PCR assays for genotyping

In the reverse RP-PCR (Figure 1), we used a locus-specific fluorescently labeled forward primer (5’-AGTACTCGCTGAGGGTGAAC-3’), a first reverse primer (5’-CGTACGCATCCCAGTTTGAGAGCCCCGGCCCCGGCCCCGGCCCC-3’) consisting of the reference amount of GGGGCC repeat and an anchor; and a second reverse primer (5’-CGTACGCATCCCAGTTTGAGA-3’) consisting of the anchor. We used the Expand Long Template PCR System (Roche Diagnostics) amplification protocol as previously described [Gijselinck et al., 2012] with an annealing temperature of 55°C.

For the STR-PCR we used a fluorescently labeled primer pair flanking the repeat (5’-CAGGTGTGGGTTTAGGAGGT-3’ and 5’-CCAGCTTCGGTCAGAGAAAT-3’) (Figure 1). The PCR amplification protocol was optimized for alleles with intermediate repeat length because of higher GC content. We used the KAPA HiFi HotStart DNA Polymerase with dNTPs kit (Kapa Biosystems) with KAPA HiFi GC buffer, 1M betaine, and 0.3µM of primers. The PCR cycling profile was as follows: denaturation at 95°C for 5 minutes, 33 cycles at 98°C for 20 seconds, 59°C for 15 seconds, and 72°C for 1 minute; and a final extension at 72°C for 5 minutes.

The resulting PCR products were size separated and analyzed on an ABI 3730 automated sequencer (Applied Biosystems) with GENESCAN LIZ600 as a size standard (Applied Biosystems) and genotypes were assigned using an in-house developed TracI genotyping software (http://www.vibgeneticservicefacility.be).

Sequencing of the *C9orf72* GC-rich low complexity sequence

We used the product of an alternative forward repeat-primed PCR (RP-PCR for sequencing; Figure 1) amplified with the Expand Long Template PCR System (Roche Diagnostics) as previously described [Gijselinck et al., 2012] with a different locus-specific reverse primer (5’-ATGCGTCGAGCTCTGAGGAG-3’). PCR amplicons were purified with 10U exonuclease I (USB Corporation) and 2U shrimp alkaline phosphatase (USB Corporation) and sequenced with the reverse primer using the BigDye Terminator Cycle Sequencing kit v3.1 (Applied Biosystems) and analyzed on an ABI 3730 DNA Analyzer (Applied Biosystems).

The Flanders-Belgian patient (N=317) and control (N=752) cohorts and 57 expansion carriers and 114 non-expansion carriers of the European cohort were successfully screened. Co-segregation of variations in the flanking repeat with the presence of a pathological G_4C_2 expansion was analyzed in two available families.

Exon sequencing

Both cohorts were screened for coding and splice-site mutations in all coding exons and exon-intron boundaries of *C9orf72*. Primers were designed with Primer 3 [Rozen and Skaletsky, 2000] (available upon request). Standard PCRs on 20ng genomic DNA amplified exons and exon-intron boundaries with optimized conditions. PCR amplicons were purified using ExoSAP-IT® (USB Corporation, Cleveland, Ohio), sequenced in both directions using BigDye® Terminator Cycle Sequencing kit v3.1 (Applied Biosystems) and analyzed on an ABI3730xl DNA Analyzer (Applied Biosystems). Sequences were analyzed using NovoSNP software [Weckx et al., 2005]. Exons containing rare mutations were also PCR sequenced in genomic DNA of 400 control individuals.

C9orf72 exon-based dosage analysis

We screened the Flanders-Belgian cohort for *C9orf72* exonic deletions or duplications using the Multiplex Amplicon Quantification (MAQ) technique [Kumps et al., 2010], consisting of a multiplex PCR amplification of fluorescently labeled test and reference amplicons, followed by fragment analysis on an ABI 3730 DNA analyzer (Applied Biosystems). Thirteen test amplicons located in all exons of *C9orf72* and 16 reference amplicons located at randomly selected genomic positions outside known copy number variants (CNVs) were simultaneously PCR-amplified on 20 ng genomic DNA. Peak areas of the test amplicons were normalized to these of the reference amplicons. Comparison of normalized peak areas between a patient and control individuals resulted in a dosage quotient (DQ) for each test amplicon, calculated by the MAQ software package (http://www.vibgeneticservicefacility.be/MAQ.htm). DQ values below 0.75 or above 1.25 were considered indicative of a heterozygous deletion or duplication respectively.

Luciferase reporter assays

We selected a 2kb genomic *C9orf72* promoter fragment (chr9:27,572,414-27,574,451; NCBIBuild37 – hg19) containing the G_4C_2 repeat and enriched for histone marks, DNaseI hypersensitivity clusters and transcription factor binding sites based on ENCODE transcription data [Gijselinck et al., 2012]. The DNA fragment was obtained by PCR amplification of DNA of individuals carrying each a different number of repeat units within the normal range (2, 9, 17, 24 units) using primers with flanked attB-sites. PCR products were cloned into the pDONR 221 vector (Invitrogen) by a standard BP recombination reaction catalyzed by the BP clonase II enzyme mix (Invitrogen) and the integrity of all inserts was confirmed by sequence analysis with the BigDye Terminator v3.1 Cycle Sequencing Kit (Applied Biosystems), with use of vector specific primers. The correct entry clones were selected and consequently cloned into the in-house developed promoterless destination vector containing the *Gaussia* luciferase reporter gene downstream of a Gateway cassette, by use of the LR recombination reaction catalyzed by the LR clonase II enzyme mix (Invitrogen).

Human HEK293T embryonic kidney cells were propagated in D-MEM (Invitrogen), with 10% fetal bovine serum, 2 mM L-glutamine, 200 IU/ml penicillin, 200 g/ml streptomycin and 0.1 mM nonessential amino acids. For transient transfection, HEK293T cells were seeded in 24-well tissue-culture dishes, at 2.10^5 cells per well, and were allowed to recover for 24 h. Cells were co-transfected with 40 ng of pSV40-CLuc plasmid that encodes the *Cypridina* luciferase gene under the control of the constitutive SV40 promoter (New England Biolabs) and 1000 ng of either one of the *C9orf72* promoter constructs, with use of 2.4 µl Lipofectamine 2000 (Invitrogen), in duplo and with three independently prepared constructs per unit.

After 24h of culturing the transfected cells, Gaussia luciferase activities (LA_G) and *Cypridina* luciferase activities (LA_C) were measured in duplo in the growth medium by use of a BioLux *Gaussia* and *Cypridina* Luciferase Assay Kit (New England Biolabs) and a Veritas Microplate Luminometer with Dual Reagent Injectors Luminometer (Promega). To correct for transfection efficiency and DNA uptake, the relative luciferase activity (RLA) was calculated as $RLA=LA_G/LA_C$. This experiment was repeated three times resulting in 36 measurements for each construct.

Relative luciferase activities between constructs of different repeat lengths were calculated by a non-parametric Mann-Whitney U test.

SUPP. REFERENCES

Gijselinck I, Van Langenhove T, van der Zee J, Sleegers K, Philtjens S, Kleinberger G, Janssens J, Bettens K, Van Cauwenberghe C, Pereson S, Engelborghs S, Sieben A et al. 2012. A C9orf72 promoter repeat expansion in a Flanders-Belgian cohort with disorders of the frontotemporal lobar degeneration-amyotrophic lateral sclerosis spectrum: a gene identification study. Lancet Neurol 11:54-65.

Kumps C, Van RN, Heyrman L, Goossens D, Del-Favero J, Noguera R, Vandesompele J, Speleman F, De PK. 2010. Multiplex Amplicon Quantification (MAQ), a fast and efficient method for the simultaneous detection of copy number alterations in neuroblastoma. BMC Genomics 11:298.

Neary D, Snowden JS, Gustafson L, Passant U, Stuss D, Black S, Freedman M, Kertesz A, Robert PH, Albert M, Boone K, Miller BL et al. 1998. Frontotemporal lobar degeneration: a consensus on clinical diagnostic criteria. Neurology 51:1546-1554.

Rozen S, Skaletsky H. 2000. Primer3 on the WWW for general users and for biologist programmers. Methods Mol Biol 132:365-386.

Van Langenhove T, van der Zee J, Gijselinck I, Engelborghs S, Vandenberghe R, Vandenbulcke M, De Bleeckere J, Sieben A, Versijpt J, Ivanoiu A, Deryck O, Willems C et al. 2012. Distinct clinical characteristics of *C9orf72* expansion carriers compared to *GRN, MAPT* and non-mutation carriers in a Flanders-Belgian FTLD cohort. Arch Neurol.

Weckx S, Del Favero J, Rademakers R, Claes L, Cruts M, De Jonghe P, Van Broeckhoven C, De Rijk P. 2005. novoSNP, a novel computational tool for sequence variation discovery. Genome Res 15:436-442.

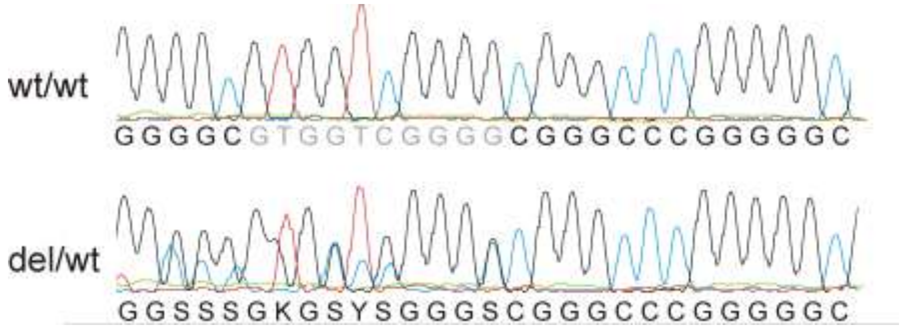

Supp. Figure S1. Comparison of sequence traces of a patient expansion carrier with the heterozygous recurrent 10bp deletion (g.26747_26756del**GTGGT**CGGGG; light grey) in the LCS and a normal sequence (Table 4). DNA was amplified using a repeat-primed PCR for sequencing (Figure 1) and the amplicons were sequenced using the locus-specific primer.

Supp. Table S1. European cohort - Patients per country overall and per clinical subgroup

Country	Partner Group	FTLD	FTLD-ALS	Uncertain	Total
**Total**	**European EOD Consortium**	**781 (28)** [(4)]	**64 (17)**	**72**	**917**
**Italy** n = 372	Brescia, Brescia University	192	11		203
	Brescia, IRCCS	47	1	21	69
	Florence, FLO	84	5	6	95
	Verona, UNIVR	1 (1)			1
	Antwerp, DMG DSF [(2)]	4			4
**Germany** n = 199	Tubingen, HIH	38	8		46
	Munich, TUM	129	2		131
	Munich, LMU	2 (1)			2
	Bonn	20			20
**Portugal** n = 191	Lisbon	77	3 (1)	34	114
	Coimbra	68	3	6	77
**Sweden** n = 79	Stockholm, KI	68 (8)	6	4	78
	Antwerp, DMG DSF [(2)]	1			1
**Spain** n = 51	Barcelona, Brain bank	13 (13)	9 (9)		22
	Barcelona	22	6		28
	Antwerp, DMG DSF	1			1
**Czech Republic** n = 10	Prague, TH	5 (5)	5 (5)		10
**Belgium** n = 10 [(3)]	Liège, CRC-ULG-CHU	1			1
	Antwerp, DMG DSF [(2)]	8		1	9
**Bulgaria** n = 3	Sofia, MU		3		3
**Austria** n = 2	Vienna, MUV		2 (2)		2

Note: [(1)] The European EOD consortium was launched in August 2011 and is coordinated by Christine Van Broeckhoven and Julie van der Zee, Department of Molecular Genetics, VIB, Antwerp, Belgium. Patients with clinical diagnosis of FTLD or ALS, or indications of FTLD or ALS symptoms are listed. The 917 patients included [(2)]6 additional patients from other European countries that had been referred for clinical genetic testing to the Diagnostic Service Facility (DSF) in the VIB Department of Molecular Genetics, DMG DSF and [(3)] 10 patients from Wallonia, the French speaking part of Belgium. [(4)]Number of patients with autopsy confirmed neuropathology diagnosis is indicated between brackets behind the total and subgroup number of patients contributed per country.

Supp. Table S2. *C9orf72* pathological G_4C_2 expansion carriers calculated per partnering European EOD consortium country

Country	Total patients n(1)	Overall carriers (%)(2)	FTLD carriers (%)(2)	FTLD-ALS carriers (%)(2)
**Total**	1205	101/1205 (8.38)	71/1118 (6.35)	30/87 (34.48)
**Belgium**(3)	369	29/369 (7.86)	22/346 (6.36)	7/23 (30.43)
**Italy**	345	21/345 (6.09)	17/328 (5.18)	4/17 (23.53)
**Germany**	199	7/199 (3.52)	4/189 (2.12)	3/10 (30.00)
**Portugal**	151	10/151 (6.62)	7/145 (4.83)	3/6
**Sweden**	75	16/75 (21.33)	12/69 (17.39)	4/6
**Spain**	51	13/51 (25.49)	9/36 (25.00)	4/15 (26.67)

Note: (1)Total number of patients with a possible or probable diagnosis of disease; (2)Carrier percentages is calculated only for countries that contributed 10 or more patients to the European cohort and for subgroups with minimal 10 patients (Supp. Table S1). (3)The Belgium sample includes apart from the Flanders-Belgian cohort an additional 9 patients ascertained in Wallonia-Belgium. N.A. Not applicable, no carriers were identified in these subgroups.

Supp. Table S3. Descriptive characteristics of G_4C_2 expansion mutation carriers

	**European cohort**			**Flanders -Belgian cohort**		
**Clinical subgroup**	**Total n**	**Average onset age years ± SD (range)**	**Average duration(2) years ± SD (range) (n)**	**Total n**	**Average onset age years ± SD (range)**	**Average duration years ± SD (range) (n)**
**Total**	73	58.0 ± 7.5 (40-75)	5.3 ± 3.5 (1-14) (n=31)	28	56.0 ± 8.6 (42-71)	5.0 ± 4.0 (2-17) (n=16)
**FTLD**	50	58.1 ± 7.4 (44-75)	6.1 ± 3.6 (1-14) (n=14)	21	55.4 ± 8.3 (42-71)	6.3 ± 4.6 (2-17) (n=10)
**FTLD-ALS**	23	57.7± 7.8 (40-71)	4.7 ± 3.5 (1-14) (n=17)	7	57.9 ± 9.7 (43-69)	2.9 ± 0.7 (2-4) (n=6)

Note: (2)Average duration of disease was calculated for n pathological G_4C_2 expansion carriers with information on age at death.

Supp. Table S4. Allelic and genotypic association of *C9orf72* G_4C_2 intermediate repeats in the Flanders-Belgian patient/control cohort

Genotype	Controls		Patients		Logistic regression	
	**N**	**%**	**N**	**%**	**OR (95%CI)**	**p-value**
S	1642	77.4	445	74.9	1.137 (0.918-1.407)	0.239
I	480	22.6	149	25.1		
S/S	630	59.4	166	55.9	ref	
S/I	382	36.0	113	38.0	1.113 (0.847-1.462)	0.442
I/I	49	4.6	18	6.1	1.374 (0.776-2.434)	0.276

Note: OR = odds ratio, 95% CI = 95% confidence interval. Patients with a known mutation in an FTLD or ALS gene (including pathological C9orf72 G_4C_2 expansion carriers) were excluded from all association analyses. p-values were calculated using logistic regression analysis and were corrected for age and gender. S = short repeat alleles with < 7 repeat units; I = Intermediate repeat alleles with ≥ 7 repeat units.

Supp. Table S5. Association with rs2814707 in the Flanders-Belgian patient/control cohort: effect with and without intermediate repeat carriers

	Genotype	Controls		Patients		Logistic regression	
		**N**	**(%)**	**N**	**(%)**	**OR (95% CI)**	**p-value**
**All patients and controls**	CC	618	58.2	166	55.9	ref	
	CT	396	37.3	109	36.7	1.011 (0.768-1.331)	0.935
	TT	47	4.4	22	7.4	1.751 (1.021-3.005)	**0.042**
**Excluding homozygous intermediates**	CC	617	61.0	166	59.5	ref	
	CT	387	38.2	108	38.7	1.026 (0.778-1.351)	0.857
	TT	8	0.8	5	1.8	2.466 (0.788-7.711)	0.121

Note: OR = odds ratio, 95% CI = 95% confidence interval. ORs were calculated for all patients, and after exclusion of patients and control individuals carrying two C9orf72 intermediate repeat alleles. Patients with a known mutation in an FTLD or ALS gene (including pathological C9orf72 G_4C_2 expansion carriers) were excluded from all association analyses. p-values were calculated using logistic regression analysis and were corrected for age and gender.
