# Supplementary figures and images for: A Pan-European Study of the C9orf72 Repeat Associated with FTLD: Geographic Prevalence, Genomic Instability, and Intermediate Repeats
Source: Hum Mutat. 2012 Oct 30;34(2):363–73. doi: 10.1002/humu.22244 (PMC3638346; doi:10.1002/humu.22244)

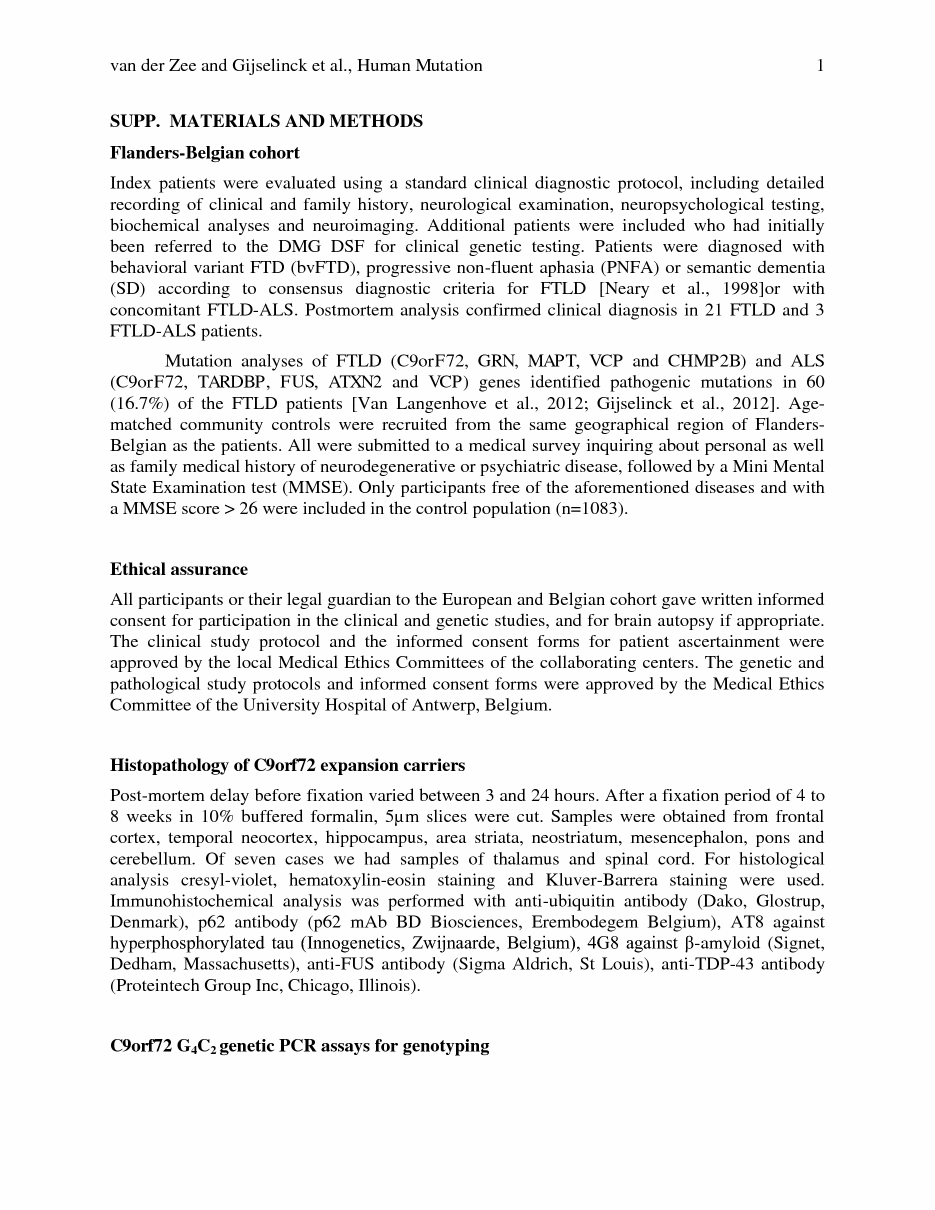

Supplement: Supplementary file 2 [file humu0034-0363-SD2.png]
